# Unlocking *n*‐alk‐1‐ynes conformers: Quantum “trigger finger” versus “stiff joint” conformations

**DOI:** 10.1002/smo2.70054

**Published:** 2026-05-06

**Authors:** Ioan Bâldea

**Affiliations:** ^1^ Theoretical Chemistry Heidelberg University Heidelberg Germany

**Keywords:** alkyne conformation, hyperconjugation, kinetic control, molecular electronics, quantum chemistry

## Abstract

Molecular conformation in *n*‐alk‐1‐ynes (CnA) is conventionally simplified to an all‐planar structure. We report a comprehensive quantum chemical analysis revealing two near‐isoenergetic rotamers at the acetylenic terminus: planar ($C_{s}$) and skewed ($C_{1}$). The high, symmetric rotational energy barrier (≈150  meV) arises from unique steric relief near the sp center coupled with electronic stabilization of $C_{1}$. This creates a unique kinetic profile: a Quantum “Trigger Finger” (α rotation) that enforces an $\approx 50\%:\;50\%\;C_{s}/C_{1}$ ensemble, sharply contrasting with the thermodynamically biased “Stiff Joint” (δ rotation) of the alkyl chain. This structural degeneracy necessitates ensemble averaging for spectroscopic data interpretation, while the slow interconversion permits kinetic trapping and intentional conformer enrichment during synthesis and molecular junction fabrication. Our work redefines the alkyne anchor, providing a blueprint for accurate interpretation of spectroscopic data and achieving conformational control in molecular electronics.

## Graphical abstract

Quantum chemical analysis reveals the unique function of *n*‐alk‐1‐yne terminus as a Quantum “Trigger Finger,” exhibiting two near‐isoenergetic conformers ( $C_{s}$ and $C_{1}$ ) locked by a symmetric high barrier. This contrasts with the lower asymmetric barrier of the conventional asymmetric “Stiff Joint” alkyl chain. The resulting ≈ 50%:50% ensemble is key for spectroscopy, yet permits kinetic trapping for predictable molecular junction fabrication.

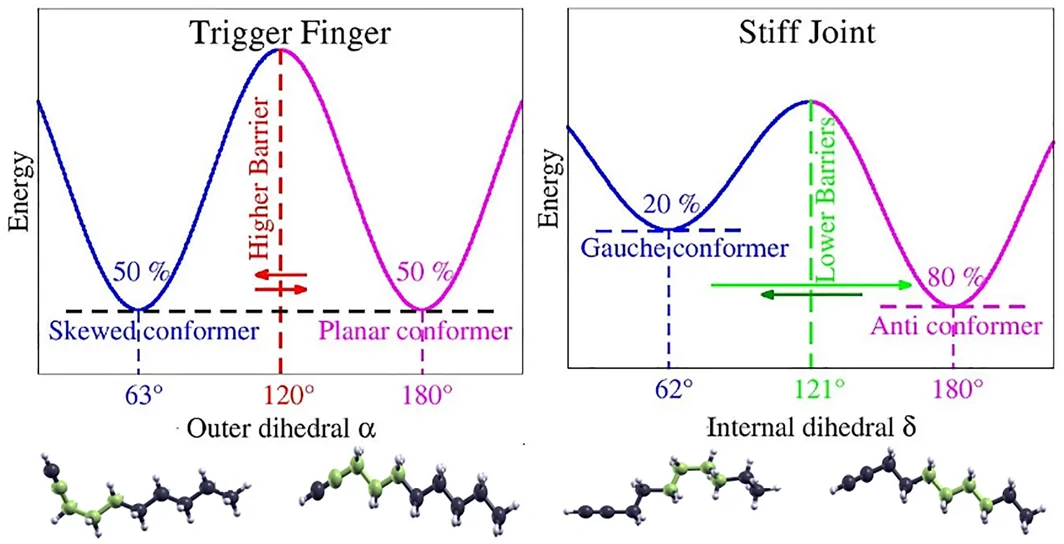

## 1 INTRODUCTION

While alkyne (C≡CH) terminals possess favorable properties (stability and strong electronic coupling) that suggest their broad use as robust anchors for molecular devices, this potential has not translated into widespread adoption. In the field of molecular junctions, thiols overwhelmingly dominate the literature,[^1^, ^2^, ^3^, ^4^, ^5^, ^6^, ^7^, ^8^, ^9^, ^10^, ^11^, ^12^, ^13^] and the use of *n*‐alk‐1‐ynes (CnA, HC≡C‐${\left({\text{CH}}_{2}\right)}_{n‐1}$‐CH3) in fabricating such devices is exceedingly rare, with only a few publications[^14^, ^15^, ^16^, ^17^] reporting CnA‐based junctions to date.

This minimal experimental adoption is coupled with a persistent theoretical blind spot: the prevailing convention assumes an all‐planar alkyne conformation ($C_{s}$), a fact reinforced by the total absence of the non‐planar $C_{1}$ conformer (to which the present study is mainly devoted) in the NIST database.^[18]^

Importantly in this context, experimental spectroscopic studies (IR, Raman, microwave) of CnA conformers in the gas phase revealing (meta)stable non‐planar conformers are limited to very short molecules: 1‐butyne (C2A),^[19]^ 1‐pentyne (C3A),[^19^, ^20^, ^21^] and 1‐hexyne (C4A).[^20^, ^22^, ^23^] These molecules are too short to function as molecular junctions; short‐circuiting issues preclude their use in fabricating robust devices. Furthermore, while those studies identified the presence of non‐planar conformers, they did not address the fundamental mechanistic question of *whether or why* the non‐planar form is stable. The crucial interplay of steric relief at the sp‐hybridized carbon and electronic stabilization via $\pi_{C\equiv C}\to \sigma_{C-H}^{*}$ hyperconjugation—an essential aspect to be discussed below—remained unexplored.

Recent transport measurements have revealed dual conductance regimes in CnA‐based junctions,^[17]^ hinting at underlying conformational complexity. However, a comprehensive quantum chemical understanding of the alkyne anchor's conformational landscape remains lacking. This fundamental gap in both experimental data and foundational structural recognition—which our investigation was triggered by—necessitates a comprehensive quantum chemical re‐evaluation of the conformational landscape of the alkyne anchor.

Our work presents a comprehensive quantum chemical analysis that resolves this long‐standing ambiguity, revealing that the acetylenic terminus acts as a unique conformational element with two stable rotamers ($C_{s}$ and $C_{1}$). The findings reported below demonstrate that the $C_{1}$ conformer is an intrinsic, kinetically persistent component of the molecular conformer ensemble, a fact vital, for example, for accurate interpretation of spectroscopic data and measurements on molecular electronic devices. First made public as a preprint^[24]^ (cited as ref. 8 in [17]), the theoretically predicted $C_{s}$/$C_{1}$ near‐degeneracy—on which the present paper elaborates—preceded, guided, and was validated by the joint theory–experiment investigation^[17]^ carried out recently in collaboration with an experimental group.

The persistent coexistence of C_*s*_ and C_1_ rotamers, locked by a high kinetic barrier, endows CnA molecules with a form of conformational memory, positioning them as *smart molecular anchors* whose interface properties can be controlled through kinetic manipulation.

## 2 RESULTS AND DISCUSSION

### 2.1 Conformational profile: The terminal α dihedral

Our investigation confirms the existence of two stable, low‐energy rotamers governed by the terminal α dihedral angle: the planar ($C_{s}$, α=180°) and the non‐planar skewed ($C_{1}$, α≈63°) conformers (Figure 1, bond metric data in Table 1).

**FIGURE 1:**
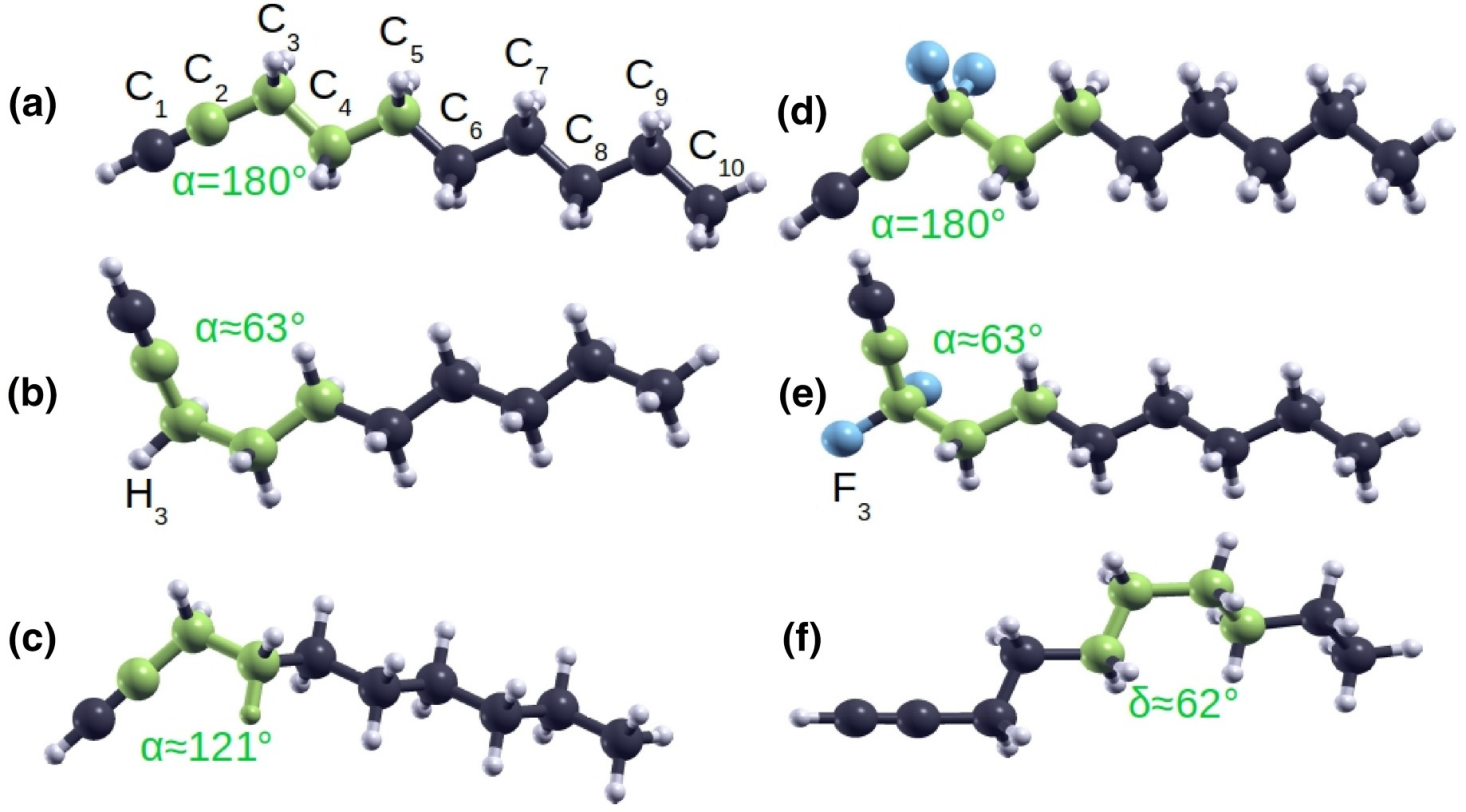
Optimized geometries for 1‐decyne (C8A) and fluorinated decyne (F2−C8A). The C atoms defining the α‐ and δ‐dihedral angles are highlighted in green. (a, b) Planar ($C_{s}$) and skewed ($C_{1}$) α‐conformers of C8A. (c) Eclipsed transition state (trans‐TS, α≈120°). (d, e) Planar ($C_{s}$) and skewed ($C_{1}$) α‐conformers of F2−C8A. (f) Non‐planar conformer with internal gauche motif (δ≈62°). IUPAC numbering is shown in panel (a).

**TABLE 1:** Bond metrics versus size for planar and skewed CnA = H‐C≡C‐${\left({\text{CH}}_{2}\right)}_{n‐1}$‐CH3 conformers optimized using the M06‐2X exchange‐correlation functional with GD3 dispersion corrections and Dunning's cc‐pVTZ basis sets.^[25]^

CnA	Conformer	Max r(H, H)	r(C_1_, C_*n*_)	r(C_1_, C_2_)	r(C_2_, C_3_)	r(C_3_, C_4_)	$∠C_{2}C_{3}C_{4}C_{5}$	$∠C_{2}C_{3}C_{4}$	$∠C_{3}C_{4}C_{5}$
C2A	Planar	5.5488	3.5208	1.1975	1.4609	1.5302	N/A	112.208	N/A
	Skewed	5.5487	3.5206	1.1975	1.4609	1.5302	N/A	112.197	N/A
C3A	Planar	6.6685	4.9566	1.1975	1.4599	1.5320	179.999	112.670	111.548
	Skewed	5.5460	3.8170	1.1977	1.4613	1.5341	62.712	112.571	112.552
C4A	Planar	7.9901	6.0336	1.1975	1.4599	1.5313	180.000	112.647	112.152
	Skewed	6.7599	5.1565	1.1977	1.4613	1.5332	63.344	112.642	113.133
C5A	Planar	9.1685	7.4223	1.1975	1.4599	1.5315	179.997	112.643	112.121
	Skewed	7.5016	5.9347	1.1977	1.4613	1.5334	63.369	112.623	113.105
C6A	Planar	10.4827	8.5677	1.1975	1.4599	1.5314	180.000	112.647	112.116
	Skewed	8.5622	7.3203	1.1977	1.4614	1.5333	63.355	112.622	113.084
C7A	Planar	11.6875	9.9286	1.1975	1.4598	1.5314	179.993	112.649	112.114
	Skewed	9.7357	8.2885	1.1977	1.4613	1.5333	63.369	112.619	113.083
C8A	Planar	12.9954	11.1065	1.1975	1.4599	1.5314	179.997	112.651	112.113
	Skewed	10.8224	9.6720	1.1977	1.4613	1.5333	63.255	112.629	113.084
C9A	Planar	14.2145	12.4497	1.1975	1.4599	1.5314	179.994	112.654	112.113
	Skewed	12.0938	10.7295	1.1977	1.4613	1.5333	63.323	112.624	113.090
C10A	Planar	15.5185	13.6474	1.1975	1.4599	1.5314	179.999	112.654	112.110
	Skewed	13.2306	12.1002	1.1977	1.4614	1.5333	63.382	112.618	113.085
C11A	Planar	16.7474	14.9789	1.1975	1.4599	1.5314	179.994	112.657	112.110
	Skewed	14.5186	13.2105	1.1978	1.4613	1.5333	63.101	112.645	113.098
C12A	Planar	18.0478	16.1896	1.1975	1.4599	1.5314	179.996	112.656	112.110
	Skewed	15.7648	14.5735	1.1977	1.4614	1.5333	63.133	112.640	113.097
C13A	Planar	19.2832	17.5125	1.1975	1.4599	1.5314	179.997	112.657	112.109
	Skewed	17.0318	15.7096	1.1978	1.4614	1.5333	63.217	112.635	113.094
C14A	Planar	20.5803	18.7320	1.1975	1.4599	1.5314	179.999	112.655	112.109
	Skewed	18.3081	17.0618	1.1977	1.4614	1.5333	63.244	112.629	113.095
C15A	Planar	21.8211	20.0488	1.1975	1.4598	1.5315	179.991	112.660	112.107
	Skewed	19.5758	18.2186	1.1977	1.4614	1.5333	63.381	112.615	113.089

Multiple density functional theory (DFT) and high‐level composite thermochemistry methods consistently confirmed that $C_{s}$ and $C_{1}$ are nearly isoenergetic, with ΔG<0.2kcal/mol (Table 2).

**TABLE 2:** Conformer stability difference $\left(\Delta G=G_{\text{skewed}}-G_{\text{planar}}\right)$ based on Gibbs free energy in kcal/mol (298 K) for n‐alk‐1‐yne molecules (CnA) spanning C2A to C15A.

CnA	G3	G4	CBS‐QB3	CBS‐4M
C2A	−0.002 (skewed)	0.000 (equal)	+0.001 (planar)	−0.006 (skewed)
C3A	−0.044 (skewed)	+0.034 (planar)	−0.009 (skewed)	−0.028 (skewed)
C4A	−0.061 (skewed)	+0.044 (planar)	−0.011 (skewed)	−0.068 (skewed)
C5A	−0.090 (skewed)	+0.029 (planar)	−0.011 (skewed)	−0.116 (skewed)
C6A	−0.100 (skewed)	+0.015 (planar)	−0.024 (skewed)	−0.149 (skewed)
C7A	−0.122 (skewed)	−0.005 (skewed)	−0.017 (skewed)	−0.142 (skewed)
C8A	−0.073 (skewed)	−0.033 (skewed)	−0.068 (skewed)	−0.158 (skewed)
C9A	−0.090 (skewed)	−0.028 (skewed)	−0.035 (skewed)	−0.142 (skewed)
C10A	−0.118 (skewed)	−0.048 (skewed)	−0.083 (skewed)	−0.164 (skewed)
C11A	−0.100 (skewed)	+0.057 (planar)	+0.034 (planar)	−0.154 (skewed)
C12A	−0.081 (skewed)	+0.063 (planar)	−0.018 (skewed)	−0.161 (skewed)
C13A	−0.109 (skewed)	0.000 (equal)	0.000 (equal)	−0.157 (skewed)
C14A	−0.142 (skewed)	0.000 (equal)	−0.027 (skewed)	−0.183 (skewed)
C15A	−0.144 (skewed)	+0.061 (planar)	−0.043 (skewed)	−0.159 (skewed)

*Note*: A positive sign indicates the planar conformer is more stable, while a negative sign indicates the skewed conformer is more stable, as confirmed by the indicator in parentheses. The minor sign changes between methods are expected for such a near‐degenerate system (|ΔG|<0.2 kcal/mol) and are within the intrinsic error limits of the composite methods. The G4 and CBS‐QB3 methods, which include more complete basis set extrapolations and higher‐order correlation treatments, are generally considered the most reliable for these subtle energetics and consistently show the skewed conformer to be slightly favored for most chain lengths.

As illustrated in Figure 2a,b, the energetic ordering is highly sensitive to the functional employed, confirming the subtle and near‐degenerate nature of the terminal conformational landscape.

**FIGURE 2:**
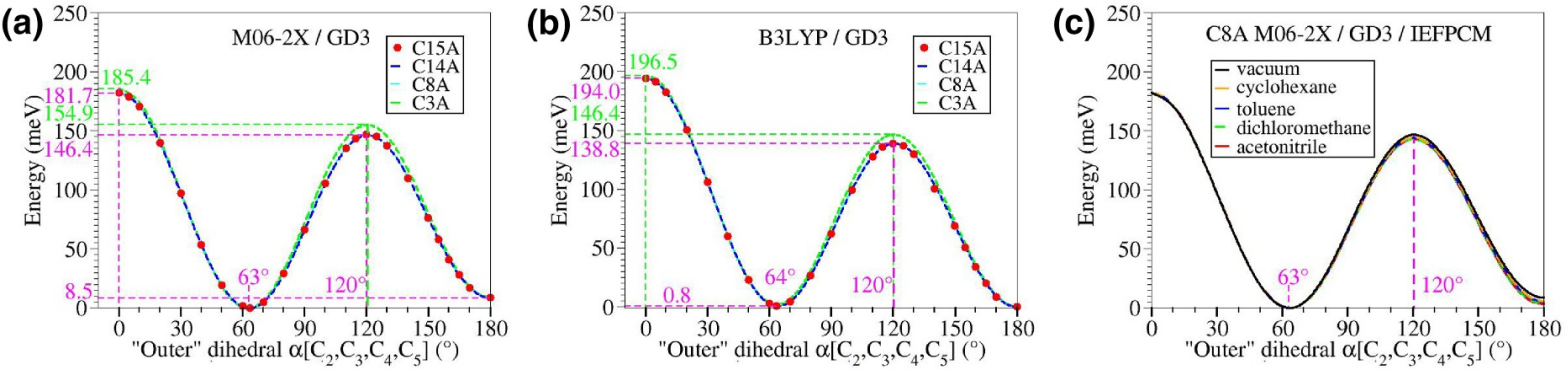
Conformational energy profile for the terminal α dihedral angle $\left(∠\left[C_{2}C_{3}C_{4}C_{5}\right]\right)$. Profiles shown are in vacuo for various chain lengths n (a) M06‐2X and (b) B3LYP, and (c) M06‐2X/IEFPCM for C8A in representative solvents. The consistent, nearly symmetric and ≈150  meV barrier separates the near‐isoenergetic planar ($C_{s}$) and skewed ($C_{1}$) minima. Solvent effects are negligible.

The two states are separated by a symmetric rotational energy barrier of approximately 150 meV (Figure 2). This barrier is consistent across chain lengths (*n*) and establishes the kinetic persistence of the individual conformers at room temperature. Furthermore, analysis using the polarizable continuum model (GAUSSIAN keyword IEFPCM^[25]^) confirmed that the barrier height and energetic degeneracy are robustly maintained in a range of representative solvents: non‐polar (cyclohexane, toluene), weakly polar (dichloromethane), and polar (acetonitrile) (Figure 2c). This validated that the rotational profile is an intrinsic molecular property driven by internal electronic and steric factors.

The identification of these two near‐isoenergetic conformers provides a quantum‐chemical foundation for interpreting experimental observations in CnA‐based systems, including recent reports of distinct transport regimes.^[17]^ The $C_{s}$/$C_{1}$ duality represents an intrinsic structural property that must be accounted for in both spectroscopic analysis and device design.

### 2.2 Ab initio wavefunction‐based validation of the rotational profile

It is well documented in quantum chemistry that, even for small molecules—including many systems of astrochemical relevance[^26^, ^27^, ^28^, ^29^]—high‐level wavefunction methods such as CCSD(T) or explicitly correlated CCSD(T)‐F12^[30]^ do not always provide conformational energy differences closer to experiment than common DFT functionals or composite thermochemistry protocols (e.g., CBS‐QB3, G4). In many cases, the latter approaches deliver more robust and balanced results, particularly for subtle effects dominated by dispersion and basis‐set incompleteness. For this reason, we consider the near‐degeneracy of the $C_{s}$ and $C_{1}$ conformers obtained from G3, G4, CBS‐QB3, and CBS‐4M calculations to be a reliable and robust prediction.

Nevertheless, to provide an independent wavefunction‐based benchmark, we carried out relaxed potential energy scans along the terminal α dihedral of 1‐decyne (C8A) using Dunning's cc‐pVTZ basis set and the following hierarchy of methods: Hartree–Fock (HF), Møller–Plesset perturbation theory to fourth order (MP2, MP3, MP4(D), MP4(DQ), MP4(SDQ)), coupled‐cluster singles and doubles (CCSD) with perturbative triples (CCSD(T)—“gold standard”), and quadratic configuration interaction singles and doubles (QCISD) with perturbative triples (QCISD(T)).

The resulting potential energy profiles are collected in Figure 3 and fully confirm the key qualitative and quantitative features already obtained at lower levels of theory:

- two near‐degenerate minima exist near α=180° (planar, $C_{s}$) and α≈64−66° (skewed, $C_{1}$);
- the barrier maximum is located near the eclipsed geometry (α≈120°);
- the classical barrier height is consistently ∼135–145 meV across correlated wavefunction methods, in excellent agreement with the value obtained with M06‐2X and composite thermochemistry approaches.

**FIGURE 3:**
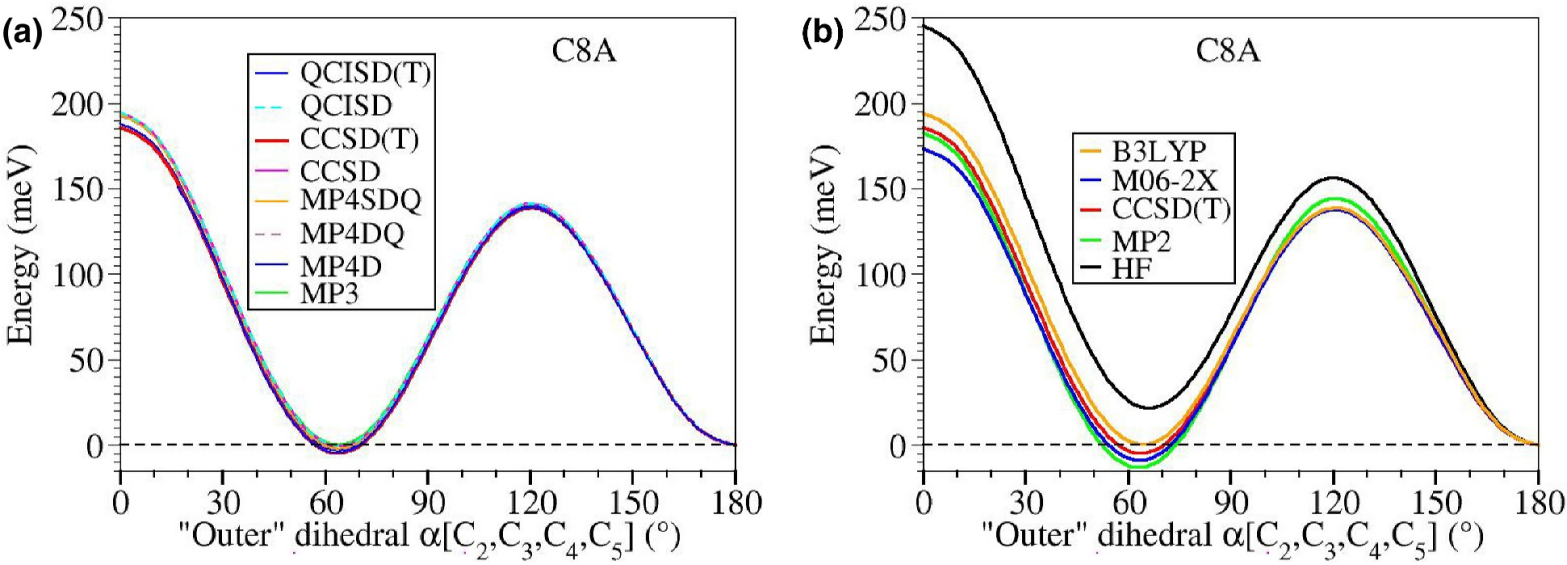
Benchmarking the conformational energy profile of 1‐decyne (C8A). The relative energy (referenced to the planar $C_{s}$ minimum at α=180°) is shown as a function of the terminal α dihedral angle $\left(∠\left[C_{2}C_{3}C_{4}C_{5}\right]\right)$. (a) High‐level wavefunction methods computed with the cc‐pVTZ basis set. The differences between the various post‐Hartree–Fock methods (MP3, MP4(D), MP4(DQ), MP4(SDQ), CCSD, CCSD(T), QCISD, QCISD(T) as implemented in GAUSSIAN 16^[25]^) are negligible—considerably smaller than the standard threshold for “chemical accuracy” (∼1 kcal/mol). Numerical values for the stationary points are provided in Table 3. (b) Comparison of MP2, M06‐2X, and B3LYP with the CCSD(T) reference. MP2 and the two DFT functionals show reasonably small deviations from the CCSD(T) benchmark, whereas the Hartree–Fock (HF) curve deviates significantly (see Table 3 for quantitative data).

Correlated methods from MP3 upward place the two minima within ≈5 meV of each other, with most favoring the skewed structure. This confirms the near‐degeneracy already observed in the composite thermochemistry protocols (G3, G4, CBS‐QB3, CBS‐4M). The minor method‐to‐method variations—both here and in the composite methods (cf. Tables 2 and 3 and Figure 3a)—are well within the standard threshold for “chemical accuracy” (∼1 kcal/mol) and are typical for subtle conformational energy differences arising from the competition between hyperconjugation and dispersion‐like correlation effects.

**TABLE 3:** Selected relative energies in meV (kcal/mol) (referenced to the planar $C_{s}$ minimum at α=180°) at key stationary points along the terminal α dihedral of 1‐decyne (C8A) in vacuo.

Method	Energy(α=0°)	$\alpha_{\text{skewed}}\left({}^{\circ}\right)$	Energy (skewed)	$\alpha_{\text{barrier}}\left({}^{\circ}\right)$	Energy (barrier $C_{s}\to C_{1}$)
HF	245.1 (5.65)	66.0	21.8 (0.50)	120.1	156.4 (3.6)
MP2	182.5 (4.21)	63.1	−12.9 (−0.30)	120.7	144.4 (3.3)
MP3	193.8 (4.47)	64.0	0.4 (0.01)	120.3	140.8 (3.2)
MP4D	187.9 (4.33)	63.8	−3.1 (−0.07)	120.4	139.6 (3.2)
MP4DQ	192.3 (4.44)	64.0	−2.4 (−0.06)	120.5	141.1 (3.2)
MP4SDQ	193.1 (4.45)	64.0	−1.9 (−0.04)	120.5	141.1 (3.2)
CCSD	194.4 (4.48)	64.1	−0.6 (−0.01)	120.4	141.2 (3.2)
CCSD(T)	185.9 (4.29)	63.7	−4.6 (−0.11)	120.4	138.8 (3.2)
QCISD	194.5 (4.49)	64.1	−0.5 (−0.01)	120.4	141.1 (3.2)
QCISD(T)	185.8 (4.29)	63.7	−4.5 (−0.10)	120.4	138.7 (3.2)
M06‐2X/GD3	173.4 (4.00)	63.3	−8.5 (−0.20)	120.5	138.0 (3.1)
B3LYP/GD3	194.1 (4.48)	64.3	0.8 (0.02)	120.6	138.8 (3.2)

*Note*: Results obtained via relaxed potential energy scans with the indicated quantum chemical methods and cc‐pVTZ basis set. The trans‐eclipsed (anti‐eclipsed) geometry (α≈120°) constitutes the relevant transition state governing the interconversion between the planar $\left(C_{s}\right)$ and skewed $\left(C_{1}\right)$ minima. The cis‐eclipsed (syn‐eclipsed) conformation (α=0°) is a higher‐energy secondary maximum of limited practical significance for the $C_{s}⇌C_{1}$ dynamics.

Abbreviations: CCSD, coupled‐cluster singles and doubles; CCSD(T), CCSD with perturbative triples; HF, Hartree–Fock; QCISD, quadratic configuration interaction singles and doubles; QCISD(T), QCISD with perturbative triples.

Among wavefunction‐based methods, only HF deviates significantly, predicting a skewed $C_{1}$ conformer that is +21.8 meV higher in energy than the planar form. This underscores the importance of dynamical correlation for accurately capturing the competition between hyperconjugation and dispersion‐like effects. Notably, the DFT functionals M06‐2X and B3LYP perform well, deviating from the CCSD(T)/QCISD(T) benchmarks by ≈4.5 meV, compared to ∼8 meV for MP2 (Table 3 and Figure 3b).

### 2.3 Mechanism and kinetics of terminal α‐dihedral interconversion

In response to the need for a clearer mechanistic picture and a more quantitative kinetic interpretation of the α≈120° barrier/trans‐transition state (trans‐TS), we characterize here the relevant transition state and estimate the interconversion dynamics, including a simple assessment of tunneling contributions.

The rotational energy profiles shown in Figure 2 reveal a direct, one‐dimensional torsional mechanism for interconversion between the planar $C_{s}$ (α=180°) and skewed $C_{1}$ (α≈63°–66°) conformers. The kinetically relevant transition state corresponds to the trans‐eclipsed (anti‐eclipsed) geometry (trans‐TS, α≈120°), where the $C_{3}-H$ bonds are eclipsed relative to the triple bond axis. Frequency calculations at the M06‐2X/GD3/cc‐pVTZ level confirm this is a transition state (trans‐TS), with a single imaginary frequency of ≈−94 c$m^{-1}$ in vacuo that corresponds predominantly to the α‐torsional coordinate. As listed in Table 4, this value is essentially invariant across a range of solvents (cyclohexane, toluene, dichloromethane, acetonitrile), mirroring the solvent robustness of the classical barrier height itself.

**TABLE 4:** Imaginary frequencies (${\text{cm}}^{-1}$, magnitude) of the two TS along the α dihedral of 1‐decyne (C8A), computed at the M06‐2X/cc‐pVTZ/GD3 level (IEF‐PCM for solvents).

Environment	$\left|\nu^{‡}\right|$ (α≈120°)	$\left|\nu^{‡}\right|$ (α=0°)
Vacuo	94.4	158.4
Cyclohexane	93.9	158.0
Toluene	93.9	158.0
Dichloromethane	93.8	158.7
Acetonitrile	93.8	159.4

*Note*: The trans‐eclipsed geometry (trans‐TS, α≈120°) is the relevant trans‐TS for conformer interconversion; the cis‐eclipsed geometry (cis‐TS, α=0°) is a higher secondary maximum.

Abbreviation: TS, transition states.

For comparison, the higher‐energy cis‐eclipsed (syn‐eclipsed) conformation (α=0°) also exhibits a single imaginary frequency (≈−158 to −159 c$m^{-1}$), but this point lies significantly above the primary barrier (∼170–250 meV depending on method) and is not on the lowest‐energy path for $C_{s}⇌C_{1}$ interconversion.

The rate constant for $C_{s}⇌C_{1}$ interconversion was estimated using the Eyring equation from transition state theory:

(1) $$k\left(T\right)=\frac{k_{B}T}{h}\exp\left(-\frac{\Delta G^{‡}}{RT}\right),$$

where $\Delta G^{‡}\approx 145$  meV (≈3.4  kcal/mol) is the Gibbs free energy barrier at 298 K (entropy of activation assumed small for this rigid torsional mode). This yields $k\approx 10^{8}$–$10^{9}$ $s^{-1}$, corresponding to mean conformer lifetimes of ∼1–10 ns at room temperature.

To demonstrate that quantum tunneling corrections are negligible under ambient conditions, we applied the simple Wigner approximation for the tunneling transmission coefficient:

(2) $$\Gamma \left(T\right)=1+\frac{1}{24}{\left(\frac{h|\nu^{‡}|}{k_{B}T}\right)}^{2},$$

using the imaginary frequency of the relevant transition state ($|\nu^{‡}|\approx 94$ c$m^{-1}$). At T=298  K this gives Γ≈1.009 (i.e., a mere ∼0.9% enhancement). Even using the larger value for the cis‐TS (∼158 c$m^{-1}$) yields Γ≈1.025 (∼2.5% enhancement), which remains insignificant compared to the overall rate uncertainty arising from the small variations in barrier height across methods (∼135–156 meV). Thus, quantum tunneling plays no meaningful role in the interconversion kinetics at room temperature.

The resulting timescale is sufficiently fast for rapid equilibration in gas phase or solution on typical experimental timescales, yet slow enough relative to ultrafast spectroscopic probes (fs–ps) and many nuclear magnetic resonance acquisition windows to allow the two conformers to coexist as a persistent ≈50%:50% ensemble. At the same time, the high barrier enables kinetic trapping during low‐temperature synthesis, flash deposition, or device fabrication protocols,^[17]^ providing a microscopic basis for the observed dual conductance regimes in CnA‐based molecular junctions.

These kinetic characteristics—rapid equilibration on laboratory timescales combined with high‐barrier persistence—underpin the unique behavior of the alkyne terminus as a “Quantum Trigger Finger” (see below).

### 2.4 Structural and electronic origin of non‐planarity

The existence and near‐degeneracy of the non‐planar $C_{1}$ state is dictated by a unique interplay of steric relief and electronic stabilization at the acetylenic terminus.

*Steric relief at the* α *‐carbon*: The core structural difference lies in the nature of the rotating bonds. While the internal δ‐rotation (Figure 4) involves an ${sp}^{3}-{sp}^{3}\;{CH}_{2}-{CH}_{2}$ bond, where the steric penalty (≈20  meV) arises from the clash between the four hydrogen atoms on the two δ carbons, the terminal α‐rotation is fundamentally an $sp-{sp}^{3}$ rotation (C2−C3). The steric environment is entirely different because the sp‐hybridized carbon (C2) is not bonded to any hydrogen atoms that could participate in a local gauche clash. This dramatic structural change provides significant steric relief, effectively suppressing the traditional gauche penalty. With the steric cost eliminated, the stability of the non‐planar $C_{1}$ state is governed by favorable electronic effects, specifically $\pi_{C\equiv C}\to \sigma_{C‐H}^{*}$ hyperconjugation, allowing it to achieve energy comparable to the planar $C_{s}$ state.

**FIGURE 4:**
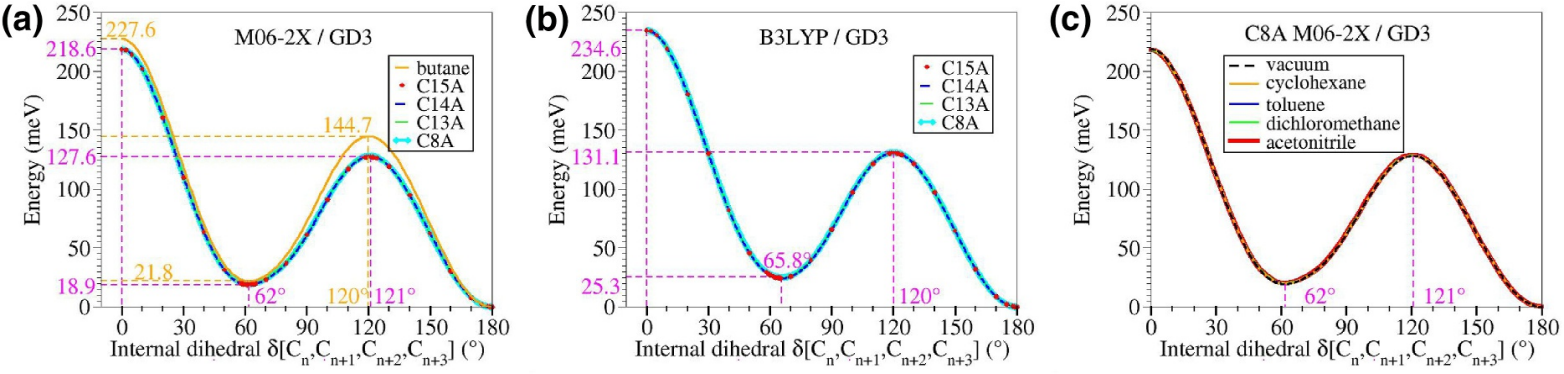
Conformational energy profile for the internal δ dihedral angle $\left(∠\left[C_{k}C_{k+1}C_{k+2}C_{k+3}\right]\right)$ along the alkyl backbone. Profiles shown are in vacuo for various chain lengths n (a) M06‐2X and (b) B3LYP, and (c) M06‐2X/IEFPCM for C8A in solvents. The profile shows a thermodynamic preference for the anti (planar) state (≈20  meV) and an asymmetric barrier (≈110  meV vs. ≈130  meV), significantly lower than the symmetric α‐barrier (Figure 2).

*Validation via fluorination*: To further probe the mechanistic origin of the $C_{s}/C_{1}$ near‐degeneracy, the fluorinated analog, 2,2‐difluoro‐*n*‐oct‐1‐yne (F2‐C8A = $H-C\equiv C-{CF}_{2}-{\left({\text{CH}}_{2}\right)}_{6}-{CH}_{3}$), was studied (Table 5). Evaluation using the most reliable composite methods (G3, G4, CBS‐QB3) confirms that the near‐degeneracy persists in F2−C8A (|ΔG|<0.4kcal/mol), but the energetic ordering becomes slightly more pronouncedly skewed ($C_{1}$ favored) compared to C8A (Table 6).

**TABLE 5:** Comparison of conformational metrics for 1‐decyne (C8A = $H-C\equiv C-{\left({CH}_{2}\right)}_{7}-{CH}_{3}$) and fluorinated decyne (F2‐C8A = $H-C\equiv C-{CF}_{2}-{\left({CH}_{2}\right)}_{6}-{CH}_{3}$) computed at the M06‐2X/GD3 level of theory.^[25]^

Mol.	Conformer	r(C_1_, C_2_)	r(C_2_, C_3_)	r(C_3_, C_4_)	r(C_1_, C_10_)	d($X_{3}$,M)	$∠X_{3}{MC}_{2}$	α
C8A	Planar	1.1975	1.4599	1.5314	11.1065	2.6302	22.514	179.997
F2‐C8A	Planar	1.1942	1.4714	1.5134	11.0756	2.8271	26.169	179.999
C8A	Skewed	1.1977	1.4613	1.5333	9.6720	2.6239	22.884	63.256
F2‐C8A	Skewed	1.1943	1.4722	1.5120	9.7052	2.8242	26.489	60.855

*Note*: $X_{3}$ is the H or F atom closest to the center (M) of the triple bond $C_{1}\equiv C_{2}$.

**TABLE 6:** Conformational stabilities for 1‐decyne (C8A = $H-C\equiv C-{\left({CH}_{2}\right)}_{7}-{CH}_{3}$) and fluorinated decyne (F2‐C8A = $H-C\equiv C-{CF}_{2}-{\left({CH}_{2}\right)}_{6}-{CH}_{3}$) using different compound chemistry models.^[25]^

Method	ΔG C8A	ΔG F2‐C8A
G3	−0.073	−0.344
G4	−0.033	−0.178
CBS‐QB3	−0.068	−0.315
CBS‐4M	−0.158	−0.113

*Note*: Differences in Gibbs free energy (ΔG = $G_{\text{torsioned}}-G_{\text{planar}}$) are given in kcal/mol. The negative values indicate the torsioned (skewed) rotamer is more stable.

This confirms the equilibrium is exquisitely sensitive to the electronic environment, as expected for a ΔG≈0 system. The substitution provides three key structural and electronic validations:

- Steric test passed: Despite the larger size of the fluorine atom, the near‐zero ΔG is maintained, confirming the sp hybridization fundamentally nullifies the steric cost of the non‐planar conformation.
- Electronic sensitivity confirmed: The C2−C3 single bond lengthens upon fluorination (e.g., $1.4613\;\overset{{}^{\circ}}{A}$ in C8A to $1.4722\;\overset{{}^{\circ}}{A}$ in F2−C8A, cf. Table 5). This lengthening indicates the powerful inductive effect of the ${\text{CF}}_{2}$ group strongly influences the σ‐framework.
- Compensatory electronic balance: While the hyperconjugative stabilization of $C_{1}$ is weakened by the C−F substitution (distance d(F3,M) larger than d(H3,M), cf. Table 5), the overall ΔG remains near zero. This outcome reveals a critical electronic balance: the loss of C−H…π stabilization is effectively compensated by the strong inductive withdrawal of the ${\text{CF}}_{2}$ group. The $C_{s}/C_{1}$ balance is thus confirmed to be the result of competing electronic forces that are highly tunable by substitution.

### 2.5 Kinetic contrast: Quantum “trigger finger” versus “stiff joint”

The CnA system's kinetic profile is defined by the stark contrast between the terminal α rotation and the internal ${sp}^{3}-{sp}^{3}\;\delta$ rotation. The comparison is best described by the analogy of a binary, kinetically locked switch versus a conventionally biased joint.

*The* α *dihedral: The kinetic toggle switch (“trigger finger”)*: The behavior of the α dihedral (C≡C−C−C) in *n*‐alk‐1‐ynes can be described as a “Trigger Finger” (*tenosynovitis stenosans*): a medical condition where a tendon catches on its protective sheath, causing the finger to lock abruptly in a bent position. This mechanism captures the essential kinetic nature of the α‐dihedral locking a binary state. The switch's function is defined by:

- The states (near‐degeneracy): The minimal energy difference (|ΔE|≤0.2kcal/mol) makes the planar ($C_{s}$) and staggered ($C_{1}$) states practically isoenergetic. This binary energy profile is the essence of the switch.
- The detent (≈150  meV barrier): The high ≈150  meV rotational barrier acts as the molecular kinetic lock (the “detent”). Since this energy significantly exceeds thermal energy (∼25  meV at room temperature), interconversion is suppressed, enforcing an abrupt, switch‐like behavior rather than continuous rotation.

The resulting nearly symmetric rotational energy barrier defines the chemical and kinetic behavior of the alkyne terminus, leading to the key feature of an ≈50%:50% $\left(C_{s}:C_{1}\right)$ equilibrium mixture in gas or solution.

*The* δ *dihedral: The stiff joint*: In contrast, the internal ${sp}^{3}-{sp}^{3}\;\delta$ dihedral is analogous to a Stiff Joint in the human body (like a knee or elbow) that has a clear, built‐in energetic preference for the extended position:

- The preferred state (built‐in energy penalty): The anti (δ=180°) state is the global minimum. The gauche (δ≈62°) state is intrinsically less stable by ≈20  meV due to the steric clash between the four ${CH}_{2}$ hydrogen atoms.
- The asymmetric resistance: The resulting rotational barrier is asymmetric (≈110  meV for gauche→anti vs. ≈130  meV for anti→gauche). This energy difference reflects the inherent, thermodynamic preference for the anti state, confirming it is a conventional alkyl chain rotamer that is always biased toward linearity, resulting in a typical ≈80% (anti) versus ≈20% (gauche) distribution at room temperature.

### 2.6 Implications: Ensemble analysis and conformational control

The persistent $C_{s}/C_{1}$ co‐existence is an intrinsic, kinetically accessible feature of the acetylenic anchor that mandates a dual approach for its application.

- Spectroscopic necessity and ensemble average: The stable equilibrium mixture is ≈50%:50%. Consequently, any measurement reflecting the state of the ensemble (e.g., standard gas‐ and solution‐state spectroscopic data) must be interpreted as the ensemble average of these two distinct conformers. This necessitates that data analysis explicitly attempt to separate and identify the contributions from both $C_{s}$ and $C_{1}$.
- Kinetic enrichment and molecular junctions: The slow $C_{s}⇋C_{1}$ interconversion rate permits significant kinetic trapping. By utilizing an appropriate synthesis pathway and preserving the product at low temperatures, one can relatively facilely enrich the mixture, leading to an intentional imbalance in the $C_{s}/C_{1}$ ratio. This enriched conformational imbalance can be preserved when the molecule is integrated into a device, such as in the fabrication of molecular junctions or self‐assembled monolayers, allowing for targeted studies where the impact of a non‐50%:50% distribution (the setting of the “Trigger Finger”) can be analyzed.

## 3 CONCLUSION

The present study represents a clear‐cut example of theory's predictive power driving subsequent experimental discovery. First made public as a preprint^[24]^ (ref. 8 in [17]), the quantum‐chemical prediction of two nearly isoenergetic $C_{s}$/$C_{1}$ conformers was the key theoretical insight that preceded, guided, and was validated by the recent experimental observation of dual conductance regimes in CnA‐based molecular junctions.^[17]^

The conformational bistability revealed in this paper positions n‐alk‐1‐ynes as *smart molecules* with intrinsic binary switching capability at the anchor interface.

The insights gained in this work into the origin and stabilization of the non‐planar alkyne terminus are essential for interpreting spectroscopic data, rationalizing the reactivity of alkynes, and contributing to their improved functionality as anchoring groups in molecular electronic devices. We establish the terminal $C_{2}-C_{3}$ bond as a discretely switchable, kinetically locked element, elevating the *n*‐alk‐1‐yne anchor to a sophisticated structural motif.

By elucidating the quantum‐mechanical origin of the $C_{s}$/$C_{1}$ near‐degeneracy and its kinetic persistence, this work provides the theoretical foundation needed to rationalize and ultimately control conformation‐dependent properties in alkyne‐terminated molecular systems.

## CONFLICT OF INTEREST STATEMENT

The author declares no conflicts of interest.

## ETHICS STATEMENT

No animal or human experiments were involved in this study.

## Data Availability

Data available on reasonable request from the author.
